# Periparturient lipolysis and oxylipid biosynthesis in bovine adipose tissues

**DOI:** 10.1371/journal.pone.0188621

**Published:** 2017-12-05

**Authors:** G. Andres Contreras, Clarissa Strieder-Barboza, Jonas de Souza, Jeff Gandy, Vengai Mavangira, Adam L. Lock, Lorraine M. Sordillo

**Affiliations:** 1 Department of Large Animal Clinical Sciences, Michigan State University, East Lansing, MI, United States of America; 2 Department of Animal Science, Michigan State University, East Lansing, MI, United States of America; University of Illinois, UNITED STATES

## Abstract

The periparturient period of dairy cows is characterized by intense lipolysis in adipose tissues (AT), which induces the release of free fatty acids (FFA) into circulation. Among FFA, polyunsaturated fatty acids are susceptible to oxidation and can modulate inflammatory responses during lipolysis within AT. Linoleic and arachidonic acid oxidized products (oxylipids) such as hydroxy-octadecadienoic acids (HODE) and hydroxy-eicosatetraenoic acids (HETE), were recently identified as products of lipolysis that could modulate AT inflammation during lipolysis. However, the effect of lipolysis intensity during the transition from gestation to lactation on fatty acid substrate availability and subsequent AT oxylipid biosynthesis is currently unknown. We hypothesized that in periparturient dairy cows, alterations in AT and plasma fatty acids and oxylipid profiles coincide with changes in lipolysis intensity and stage of lactation. Blood and subcutaneous AT samples were collected from periparturient cows at -27±7 (G1) and -10±5 (G2) d prepartum and at 8±3 d postpartum (PP). Targeted lipidomic analysis was performed on plasma and AT using HPLC-MS/MS. We report that FFA concentrations increased as parturition approached and were highest at PP. Cows exhibiting high lipolysis rate at PP (FFA>1.0 mEq/L) had higher body condition scores at G1 compared to cows with low lipolysis rate (FFA<1.0 mEq/L). Concentrations of plasma linoleic and arachidonic acids were increased at PP. In AT, 13-HODE, and 5-, 11- and 15-HETE were increased at PP compared to G1 and G2. Concentrations of beta hydroxybutyrate were positively correlated with those of 13-HODE and 15-HETE in AT. Plasma concentrations of 5- and 20-HETE were increased at PP. These data demonstrate that prepartum adiposity predisposes cows to intense lipolysis post-partum and may exacerbate AT inflammation because of increased production of pro-inflammatory oxylipids including 5- and 15-HETE and 13-HODE. These results support a role for certain linoleic and arachidonic acid-derived oxylipids as positive and negative modulators of AT inflammation during periparturient lipolysis.

## Introduction

During the last 3 weeks of gestation and until 5–6 weeks post calving, dairy cows exhibit an increased rate of lipolysis in adipose tissues (AT) that induces the release of free fatty acids (FFA) into both the AT milieu and circulation [1]. Lipolysis is a mechanism of metabolic adaptation that is necessary to offset the negative energy balance in dairy cows due to their drastic appetite reduction and copious milk production. Lipolysis triggers an AT remodeling process that is characterized by a moderate inflammatory response with immune cell infiltration and changes in extracellular matrix components [2, 3]. AT remodeling minimizes lipotoxicity induced by FFA and promotes adipogenesis, which, in turn, replenishes adipocyte populations that buffer excess FFA [4]. As lactation progresses with a return to positive energy balance, lipolysis is reduced and inflammation in AT is resolved. However, dairy cows exhibiting excessive lipolysis develop chronic AT inflammation that can perpetuate lipolysis and lead to disease [5, 6]. In fact, elevated concentrations of blood FFA, including polyunsaturated FA (PUFA), is a risk factor for the development of periparturient diseases such as ketosis, fatty liver, and metritis [7]. The mechanisms leading to uncontrolled AT inflammatory responses are unclear, but may be linked to alterations in the biosynthesis of PUFA-derived pro-inflammatory byproducts of lipolysis during the periparturient period.

Although PUFA comprise only around 2–4% of the total FA content of the bovine adipose organ [8, 9], when released during lipolysis, these molecules can modulate inflammation in AT and possibly other tissues through the actions of their oxidized byproducts (oxylipids). Oxylipids are a class of lipid mediators produced by the enzymatic and non-enzymatic oxidation of PUFA [10]. The most abundant oxylipid substrates are linoleic (LA) and arachidonic (ArA) acids. Studies in rodent models of lipolysis demonstrate that the activation of cyclooxygenase 2 (COX2) and 5- and 15- lipoxygenases (5LOX, 15LOX) during lipolytic events enhances oxylipid biosynthesis [11]. LA-derived oxylipids, including 9- and 13-hydroxyl-octadecadienoic acids (HODE), promote inflammatory responses within AT by acting as chemoattractants of AT macrophages [12]. Similarly, oxidized metabolites of ArA, including prostaglandin E_2_ (PGE_2_) and 5- and 11-hydroxyeicosatetraenoic acids (HETE), enhance adipocyte secretion of macrophage chemoattractant protein-1 during lipolysis [11]. Other oxylipids detected after lipolysis stimulation of adipocytes include the LA- and ArA-derived products of the cytochrome P450 (CyP450) expoxygenases [11]. CyP450 products derived from LA include: the epoxides 9,10- and 12,13- epoxyoctadecanoic acids (EpOME) and their downstream diols, 9,10- and 12,13-dihydroxyoctadecenoic acids (DiHOME). The latter are synthesized by the soluble epoxide hydrolase (sEH) enzyme. The ArA metabolites of CyP450 include the hydroxylation product 20-HETE, the epoxides 5,6-, 8,9-, 11,12- and 14,15- epoxyoctadecanoic acids (EET), and the sEH derived diols 8,9-, 11-12-, and 14,15- dihydroxy epoxyoctadecanoic acids (DHET). In the AT, most epoxides characterized to date have anti-inflammatory properties and reduce adipogenesis [13]. In contrast, the majority of diols described in the literature are considered pro-inflammatory [14]. Nonenzymatic oxidation of LA and ArA can also occur during lipolysis. LA-derived 9- and 13-HODE can be produced by non-enzymatic oxidation. Similarly, ArA can be oxidized non-enzymatically to yield 11-HETE and the isoprostanes 8-iso-PGF2alpha and 8,12-iso-iPF2alpha-VI and these oxylipids were described as products of lipolysis [11]. In healthy dairy cows, the plasma and milk concentrations of oxylipids from COX, LOX, CYP450, and non-enzymatic oxidation were shown to have dynamic changes as lactation progresses [15, 16]. However, oxylipid profiles in AT of periparturient cows have not been described before and the effect of lipolysis intensity on oxylipid biosynthetic pathways is currently unknown.

In the present study, we hypothesize that circulating and AT content of FA and oxylipid profiles are associated with lipolysis intensity and the time relative to calving during the periparturient period. By using targeted lipidomic analysis and assessing AT inflammatory responses, we demonstrate that FA content of AT and plasma is modified as lactation cycle advances. Parturition and the onset of lactation enhance the production of specific LA- and ArA-derived oxylipids, and their content is modified by lipolysis intensity.

## Materials and methods

### Animals and sample collection

All animal procedures were approved by the Michigan State University Animal Care and Use Committee (07/14-117-00). Nine healthy, mature, and pregnant (210–240 days of gestation) Holstein cows in their second or third lactation were selected after cessation of milking (i.e. dry-off) from the Michigan State University Dairy Teaching and Research Center. Body condition scoring was performed at selection by 3 experienced technicians using a 1–5 scale as described by [17]. Animals were housed in tie-stalls and monitored daily by experienced farm personnel throughout the last month of gestation and into early lactation. Animals were fed a pre-calving (i.e. close-up) diet for 4 weeks preceding parturition, at which point they were transitioned to a lactation diet (Tables A and B in S1 File). Blood and subcutaneous AT samples were collected at 27±7 (G1) and 10±5 (G2) d prepartum and at 8±3 (PP) d postpartum. Blood was collected in EDTA and serum separator tubes at 7:30 AM immediately before feeding and prior to AT specimen collection. EDTA blood samples were kept on wet ice until centrifugation that was performed for 20 min at 1000 × *g* at 15°C. Next, plasma and serum fractions were collected, aliquoted and stored in a −80°C freezer for further analyses. A subsample of plasma was used to quantify FFA and β-hydroxybutytyrate (BHB) as previously described [18]. Based on plasma FFA concentrations at PP, cows were assigned post hoc to high (HL, FFA>1.0 mEq/L) and low lipolysis (LL, FFA<1.0 mEq/L) groups for data analyses purposes. Of the nine cows selected for this study, only one cow in the HL group had a health event reported on day 1 after calving, retained fetal membranes, that was treated following Michigan State University Dairy Teaching and Research Center health protocols.

The AT samples (3–5 g) were obtained surgically from the tail head area through a 4-cm V-incision that was made 5 to 10 cm laterally from the medial line and 5–10 cm cranial to the base of the tail. The surgical area was anesthetized prior to the incision using 2% lidocaine hydrochloride. Subsequent AT specimens were obtained from a site opposite to the previous incision. Once collected, AT samples were divided and: a) snap frozen and stored at −80°C for proteomic and lipidomic analyses, b) immersed in RNA Later (Life Technologies, Carlsbad, CA) and stored at −80°C for transcriptomic analyses, or c) fixed in 4% paraformaldehyde (Electron Microscopy Sciences, Hartfield, PA) for histological analysis.

### Gene expression analysis

RNA was extracted from AT samples using the Maxwell® RSC simplyRNA Tissue Kit (Cat# AS1390) and a Maxwell® RSC Instrument according to manufacturer instructions (Promega, Madison, WI). In brief, 50 mg of subcutaneous AT sample were homogenized in 200 μl of chilled 1-Thioglycerol/Homogenization solution. Homogenate was added to 200 1μ of Lysis Buffer and then vortexed vigorously for 15 s. The sample (~400 μl) was then automatically processed on the Maxwell® RSC Instrument using simplyRNA cartridges containing 10 μl of blue DNase I solution in each designated well. After extraction, samples were eluted in nuclease-free water. Purity, concentration, and integrity of mRNA were evaluated using a NanoDrop 1000 spectrophotometer (Thermo Scientific, Wilmington, DE) and an Agilent Bioanalyzer 2100 system (Agilent Technologies, Santa Clara, CA). All samples had a 260:280 nm ratio between 1.9 and 2.1 and a RNA integrity number >6. Conversion to cDNA was performed using the Applied Biosystems High Capacity cDNA Archive Kit (Applied Biosystems, Foster City, CA). All quantitative qPCR assays were conducted with TaqMan gene expression primers and reagents from Applied Biosystems. Taq primers were either commercially available or designed from bovine sequences with the Applied Biosystems Pipeline software and synthesized by Applied Biosystems Inc (Table C in S1 File). Samples were assayed in triplicate on the high-throughput qPCR instrument Wafergen Smartchip System (WaferGen Biosystems, Fremont, CA). cDNA was diluted in RT-PCR grade water and loaded at a primer-efficient concentration of 714 pg/μL. A non-reverse-transcriptase control sample validated absence of genomic DNA. Reference genes were selected using GeNorm from a pool of 7 candidates (*ACTB*, *B2M*, *EIF3K*, *GAPDH*, *PPIA*, *RPL0*, *RPS9*). The set of reference genes with lowest pairwise variation value (V-value = 0.128) included eukaryotic translation initiation factor 3 subunit K (*EIF3K*), β-2 microglobulin (*B2M*), and ribosomal protein S9 (*RPS9*), and were selected for analyses purposes based on the recommended threshold cut-off V-value < 0.15 [19]. Expression of genes of interest was normalized against the geometric mean of selected reference genes as described by Hellemans et al., [20].

### Western blotting

Protein was extracted in RIPA buffer (Teknova, Hollister, CA) containing protease (Roche, San Francisco, CA) and phosphatase (Thermo Scientific, Waltham, MA) inhibitors and quantified using BCA reagent as described in [21]. Western blots were performed using the size-based Simple Western system (ProteinSimple, Santa Clara, CA) that automatically processes protein samples by capillary technology. Antibodies were from Cell Signaling Technology (Danvers, MA) and included rabbit anti-mouse hormone sensitive lipase (HSL, 1:100, #4107), rabbit anti-mouse phosphorylated HSL serine 660 (pHSL; 1:50, #4126), and rabbit anti-human vinculin as loading control (1:1,000, #4650). Digital images of the Western blots were analyzed with Compass software (ProteinSimple, Santa Clara, CA), and the quantified data for HSL and pHSL was normalized to vinculin. Data are reported as the ratio of pHSL:HSL as previously reported by [21].

### Histology

Adipocyte cell area was determined in hematoxylin- and eosin-stained sections of paraformaldehyde fixed paraffin-embedded tissue. The area of 100 adjacent cells from each of 5 randomly selected fields were measured using ImageJ software (National Institutes of Health, Bethesda, MD) as described previously [22].

### Lipidomics

Targeted oxylipids in plasma and adipose tissue of cows were quantified using liquid chromatography tandem mass spectrometry (LC/MS/MS). A full list of metabolites can be found in [23].

Chemicals: Acetonitrile, methanol, and formic acid of liquid chromatography/mass spectrometry (LC/MS) grade were purchased from Sigma-Aldrich (St. Louis, MO. USA). Deuterated and non- deuterated oxylipid standards were purchased from Cayman Chemical (Ann Arbor, MI, USA). Butylated hydroxy toluene (BHT) was purchased from ACROS (New Jersey, USA), Ethylenediaminetetraacetic acid (EDTA) and triphenylphosphine (TPP) were purchased from Sigma-Aldrich (St. Louis, MO. USA), and indomethacin was purchased from Cayman Chemical (Ann Arbor, MI, USA).

#### Lipid extraction

Plasma samples were collected, extracted, and analyzed using methods published previously by Mavangira et al., [23]. Briefly, 2 ml of flash frozen plasma was thawed on ice, mixed with an antioxidant reducing agent mixture at 4 μl/ml to prevent degradation of pre-formed oxylipids and prevent ex-vivo lipid peroxidation [24]. The antioxidant reducing agent mixture consisted of 50% methanol, 25% ethanol, and 25% water with 0.9 mM of BHT, 0.54 mM EDTA, 3.2 mM TPP, and 5.6 mM indomethacin. Samples were combined with a 15 μl mixture of internal standards containing 0.25 uM 5(S)-HETE-*d*_8_, 0.25 uM 15(S)-HETE-*d*_8_, 0.5 uM 8(9)-EET-*d*_11_, 0.5 uM PGE_2_-*d*_9_, and 0.25 uM 8,9-DHET-*d*_11._ Afterward methanol was added to yield a 60% methanol solution. Samples were vortexed two minutes, incubated at room temp for 15 min and centrifuged at 4,816 x *g* for 20 min at 4^o^ C. Supernatant was diluted with high performance liquid chromatography (HPLC) water containing 0.01% formic acid to yield a 20% methanol solution. Adipose tissue samples were weighed for normalization, then homogenized in a solution of 500 μl methanol, 2 μl antioxidant reducing agent, and 15 μl of the internal standard mixture. After a 5-minute 14,000 x *g* 4^o^ C centrifugation, the clear supernatant was transferred to a clean tube. A second liquid extraction was performed with 500 μl of methanol, the clear supernant was added to the first extract. The methanol was diluted with 9 ml of water followed by an adjustment to a less than 4 pH with HCL. Solid phase extraction for both plasma and adipose tissue was carried out with Phenomenex, Strata-X 33 μm Polymeric Reversed Phase 200 mg/6ml tubes preconditioned with 6 ml methanol followed by 6 ml HPLC water. Supernatants were loaded into the columns, then washed with 20% methanol and eluted with a 50:50 mixture of methanol and acetonitrile with 2% formic acid. Volatile solvents were removed using a Savant SpeedVac and residues were reconstituted in methanol, mixed at a 2:1 ratio with HPLC water and stored in chromatography vials at -80 ^o^C until analysis. A 6-point standard curve was created with a mix of standards and the internal standards mentioned above for quantification.

#### Liquid chromatography-tandem mass spectrometry analyses

Details of LC/MS/MS analysis can be found published by Mavangira et al., [23]. In short, the quantification of metabolites was accomplished on a Waters Xevo-TQ-S tandem quadrupole mass spectrometer using multiple reaction monitoring. Chromatography separation was performed with an Ascentis® Express C18 HPLC column, held at 50 ^o^C and autosampler held at 10 ^o^C. Mobile phase bottle A was water containing 0.1% formic acid and mobile phase bottle B was acetonitrile, the flow rate was 0.3 mL/min. Liquid chromatography separation took 15 minutes per sample. All oxylipids were detected using electrospray ionization in negative-ion mode. Cone voltages and collision voltages were optimized for each analyte using Waters QuanOptimize software and data analysis was carried out with Waters MassLynx software.

#### Liquid chromatography-tandem mass spectrometry quantification of fatty acids

All fatty acids were quantified as described by Ryman et al., [25] with some modifications. The samples injected in the LC/MS/MS system were reinjected in the LC/MS. Briefly, reverse-phase LC on a Waters Acquity UPLC utilizing a BEH C18 1.7 μ*M* (2.1 × 100 mm) column with a flow rate of 0.6 mL/min at 50°C. The quadropole MS was in electrospray negative ionization mode and voltage was −3 kV with the turbo ion spray source temperature at 450°C. The gradient mobile phase was programmed in the following manner (A/B/D ratio): time 0 to 0.5 min (30/5/65), to (65/5/30) at 1.0 min, to (85/10/5) at 5.50 min, to (89/10/1) at 7.0 min, and held until 11.5 min, then return to (30/5/65) at 11.01 min, and held at this condition until 15.0 min. In this gradient mobile phase A = Acetonitrile, B = Methanol, and D = 0.1% Formic Acid. A list of fatty acids can be found in [23]. PUFA, SFA, and MUFA were quantified by matching mass-1 and retention time with corresponding deuterated internal standard abundance and calibrated to a linear 7-point standard curve (R^2^ > 0.99). Fatty acids were quantified with Waters Empower 3 software (Waters).

### Statistical analyses

Statistical analyses were carried out using the MIXED procedure of SAS (version 9.4; SAS Institute, Cary, NC) to assess the fixed effects of time (G1, G2 and PP), lipolysis intensity (HL and LL), and their interaction. Cow was included in the model as a random effect. Repeated measures over time were modeled with an autoregressive covariance structure and denominator degrees of freedom were estimated using the Kenward-Rogers method. For adipocyte area analyses, data within time and lipolysis intensity was grouped by adipocyte size (<500, 501–1000, 1001–2000, 2001–4000, 4001–6000, 6001–8000, 8001–10000, and >10000 μm^2^) and frequencies were analyzed using a model including the fixed effect of time, lipolysis intensity, and its interaction and means compared by a Bonferroni post hoc analysis. Values were deemed outliers and omitted from analysis when Studentized residuals were > 3.0 or < −3.0. Normality of the residuals was checked with normal probability and box plots and homogeneity of variances with plots of residuals vs. predicted values. Partial correlations coefficients were assessed using the CORR procedure of SAS to determine correlations among specific FA concentrations and oxylipids in plasma and AT. Interactions were investigated when *P* < 0.15 using the slice option, and slices were declared significant at *P* < 0.05.

## Results

### Lipolysis during late gestation and the onset of lactation

**Fig 1:**
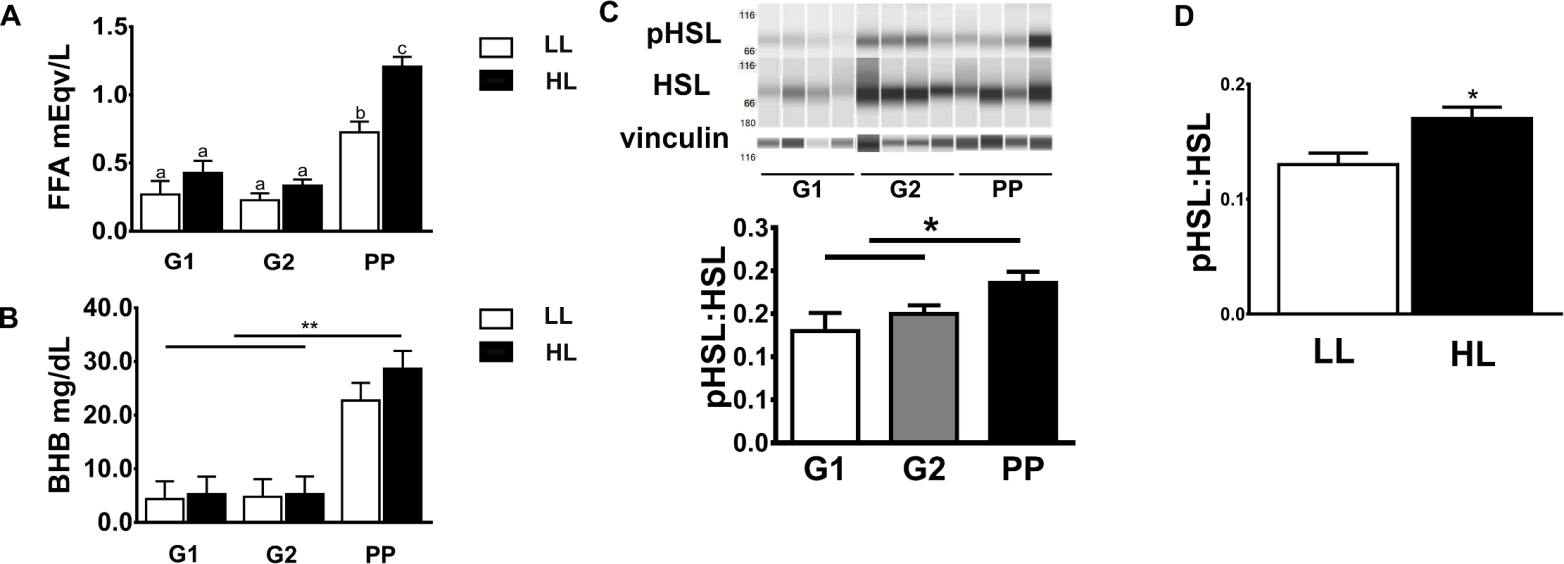
Lipolysis increases as gestation progresses and peaks after parturition. Blood and adipose tissue samples were collected from dairy cows (n = 9) at -27±7 (G1) and -10±5 d (G2) prepartum and at 8±3 d postpartum (PP). **(A)** Circulating free fatty acids (FFA); **(B)** circulating beta hydroxyutyrate (BHB); **(C)** phosphorylation ratio (at Serine^660^) of hormone sensitive lipase (HSL) in subcutaneous adipose depots from 4 different cows; **(D)** HSL phosphorylation ratio (at Serine^660^) in cows with low (LL, FFA<1.0 mEq/L, n = 4) and high (HL, FFA≥1.0 mEq/L, n = 5) lipolysis rate at PP. pHSL = rabbit anti-mouse phosphorylated HSL serine^660^. Data are means ± SEM. Significant differences are indicated by * and letters a, b, c (P<0.05), ** (P<0.01).

**Fig 2:**
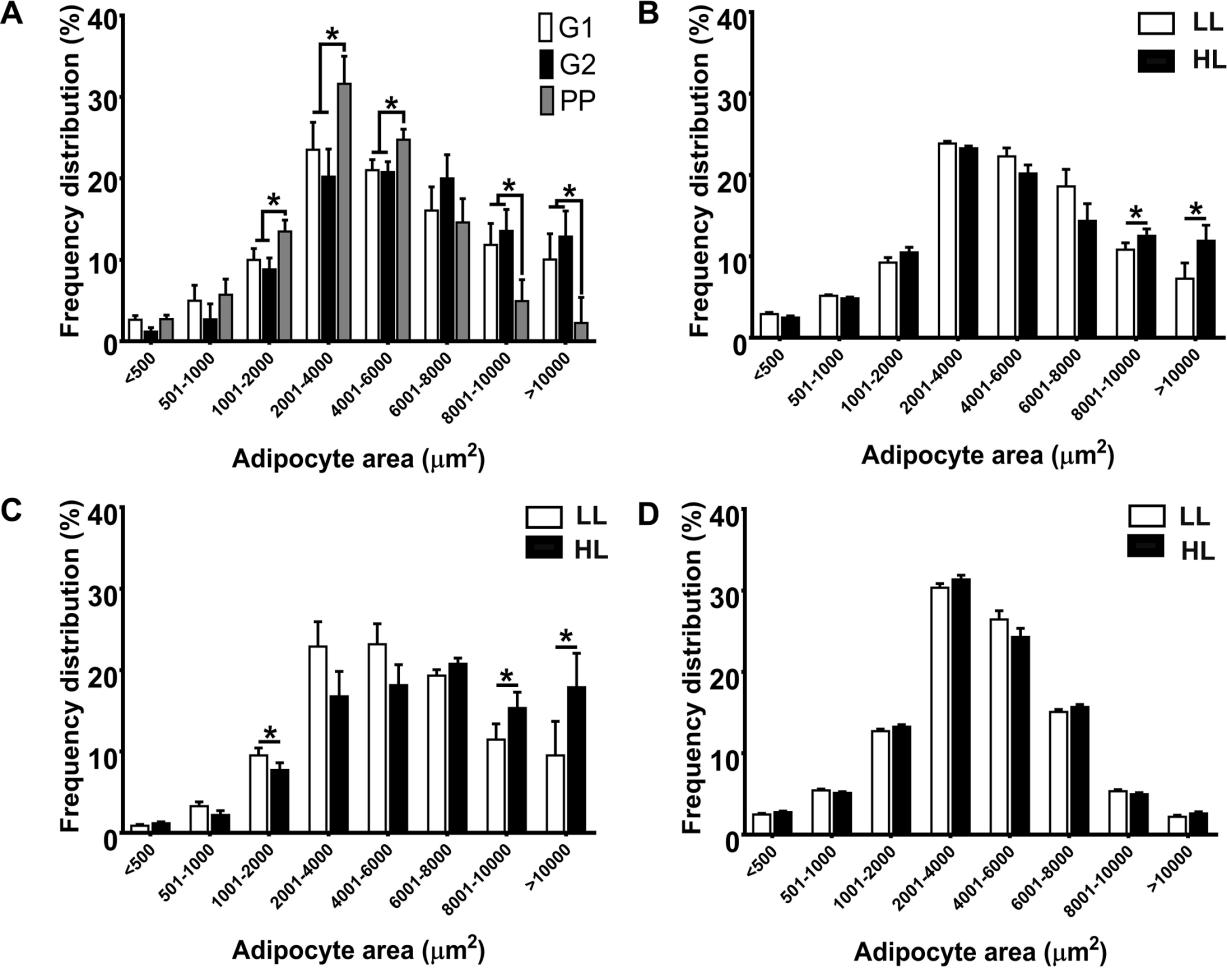
Adipocyte size distribution in subcutaneous adipose tissue (AT) is modified by the onset of lactation and lipolysis intensity in adipose tissue. (**A**) Frequency of adipocyte sizes in AT collected from dairy cows (n = 9) at -27±7 (G1) and -10±5 d (G2) prepartum and at 8±3 d postpartum (PP). Adipocyte size distribution at G1 (**B**), G2 (**C**), and PP (**D**) in cows with low (LL, FFA<1.0 mEq/L, n = 4) and high (HL, FFA≥1.0 mEq/L, n = 5) lipolysis at PP. Significant differences are indicated by * (P<0.05).

Lipolysis increased at PP, compared to G1 and G2, as reflected by elevated circulating FFA concentrations (G1: 0.22±0.05, G2: 0.27±0.05, PP:0.99±0.05 mEq/L, *P*<0.05; Fig 1A). Across sampling points, plasma FFA concentrations were greater in HL (0.65±0.05 mEq/L) compared to LL (0.40±0.06 mEq/L). Plasma BHB was increased at PP compared to G1 and G2, however, no differences were observed between HL and LL (Fig 1B). The phosphorylation of HSL at serine 660 in AT increased at PP compared to G1 and G2 (Fig 1C). Across sample time points, HSL phosphorylation was greater in HL compared to LL (Fig 1D). Adipocyte area increased between G1 (4,023±1,268 μm^2^) and G2 (4,698±1,268 μm^2^) and then was reduced at PP (3,159±1,268 μm^2^, *P*<0.01). This was due to a lower frequency of large adipocytes (>8,000) and higher number of smaller adipocytes (2,000–6,000 μm^2^) at PP (Fig 2A). HL exhibited increased frequency of large adipocytes (>8,000 μm^2^) compared to LL at G1 and G2 (Fig 2B and 2C). However, at PP the frequency distribution of adipocyte areas was similar between groups (Fig 2D). At G1, HL exhibited increased (*P*<0.05) body condition score (3.95 ± 0.19), and thus greater adiposity, than LL (3.23 ± 0.04). Blood concentrations of negative acute phase proteins that act as FFA carriers such albumin and fetuin A and cholesterol did not differ between HL and LL groups (Table D in S1 File)

### FA and oxylipids during the periparturient period

Using targeted lipidomics, we analyzed the abundance of specific FA and oxylipids in plasma and AT (Table 1). Plasma saturated FA including lauric (C12:0), myristic (C14:0), and palmitic (C16:0, *P =* 0.07) acids, increased at PP compared to G1 and G2 (Table 1). Similarly, the monounsaturated FA, palmitoleic (C16:1) and oleic (C18:1), and the PUFA, LA [C18:2 (n-6)], linolenic [C18:3 (n-3)], ArA [C20:4 (n-6)], and EPA [eicosapentanoic acid, C20:5 (n-3)], were increased in plasma at PP compared to G1 and G2 (Table 1). Across time, HL exhibited decreased plasma concentrations of C18:0, ArA, EPA, and docosahexanoic acid [DHA, C22:6 (n-3)] compared to LL (Table 1). As expected, concentrations of circulating FFA were positively associated with those of specific plasma fatty acids including: C12:0, C14:0, C16:1, C18:1, LA, C20:4 (n-6), and C20:5 (n-3) (Table 2). Similarly, circulating BHB was positively correlated with plasma C14:0, C16:1, LA, and C18:3 (n-3) (Table 2).

**Table 1:** Dynamics of circulating and adipose tissue fatty acids in periparturient dairy cows at -27±7 (G1) and -10±5 d (G2) prepartum and at 8±3 d postpartum (PP). Cows were grouped by lipolysis intensity (LI) based on FFA concentrations at PP in low (LL = FFA<1.0 mEq/L) and high (HL, FFA≥1.0 mEq/L) lipolysis rate.

		G1		G2		PP			P-value		
	Fatty acid^1^	LL	HL	LL	HL	LL	HL	SEM	Time	LI	Time by LI
**Plasma** μM/L	C12:0	14.93	15.45	17.12	12.69	26.05	37.36	6.45	<0.05	ns^2^	ns
	C14:0	44.34	74.53	62.00	59.88	214.53	201.49	39.16	<0.001	ns	ns
	C16:0	1224.89	938.23	1381.47	744.33	1820.14	2463.91	678.82	0.07	ns	ns
	C16:1	94.01	98.73	89.54	88.08	283.18	368.31	70.21	<0.001	ns	ns
	C18:0^3^	3557.45	726.56	3558.81	1357.28	2695.13	2008.17	1032.07	ns	0.07	ns
	C18:1	900.15	938.23	786.94	982.05	1652.71	1370.44	321.99	<0.05	ns	ns
	C18:2 (n-6)	2466.66	1601.05	1960.48	1679.09	4632.87	4347.09	935.71	<0.01	ns	ns
	C18:3 (n-3)	35.79	31.69	27.34	19.42	60.7	45.98	9.85	<0.01	ns	ns
	C18:3 (n-6)	21.99	10.02	14.69	10.02	25.68	22.53	6.92	ns	ns	ns
	C20:4 (n-6)	33.02	11.35	21.48	12.12	51.86	53.12	13.61	<0.05	<0.05	ns
	C20:5 (n-3)	9.01	5.10	7.23	2.80	40.02	35.43	12.17	<0.05	<0.05	ns
	C22:4 (n-6)	33.31	23.51	23.49	9.71	33.63	22.04	9.09	ns	<0.05	ns
** **	C22:6 (n-3)	10.10	3.58	6.42	1.64	11.44	3.69	2.62	ns	<0.01	ns
**Adipose Tissue** μM/μg	C12:0	590.60	209.62	177.42	154.29	214.02	311.74	117.86	ns	ns	ns
	C14:0	2980.03	2337.22	1564.67	1157.37	1610.40	3544.14	888.09	ns	ns	ns
	C16:0	73876.42	85431.07	67505.74	63714.24	24593.86	45266.58	29943.16	<0.01	ns	ns
	C16:1	8250.94	5997.26	2286.10	2215.31	2235.41	1046.43	2294.53	<0.05	ns	ns
	C18:0	17495.52	18299.78	13931.16	15049.04	18315.96	15057.37	5613.68	ns	ns	ns
	C18:1	20769.47	19602.12	16185.50	16458.85	18183.05	23331.93	3952.00	0.10	ns	ns
	C18:2 (n-6)	2216.66	1043.77	422.86	345.89	577.75	353.75	386.95	<0.05	ns	ns
	C18:3 (n-3)	371.51	292.46	197.92	141.62	92.90	121.95	34.46	<0.05	ns	ns
	C18:3 (n-6)	87.33	31.44	7.08	5.56	10.15	14.09	48.74	ns	ns	ns
	C20:4 (n-6)	56.68	32.04	10.47	7.05	22.78	13.23	30.86	0.09	ns	ns
	C20:5 (n-3)	0.11	0.33	0.09	0.05	0.15	0.19	0.45	ns	ns	ns
	C22:4 (n-6)	9.33	1.30	0.30	0.43	0.38	5.99	2.73	ns	ns	ns
	C22:6 (n-3)	0.34	0.25	0.38	0.47	0.17	0.18	0.13	ns	ns	ns

^1^ Values are least squares means of fatty acid concentrations in plasma and adipose tissue as measured in HPLC MS/MS.

^2^ ns = P > 0.10.

^3^ An outlier from LL treatment at G1was removed from C18:0 data analysis

**Table 2:** Partial correlation coefficients for circulating concentrations of free fatty acids (FFA) and β hydoxybutyrate (BHB) and contents of specific plasma FA and adipose tissue oxylipids, and gene expression of oxylipid enzymes and inflammatory markers in periparturient cows.

	FFA vs.	Correlation	*P*	BHB vs.	Correlation	*P*
**Plasma FA**	BHB	0.510	**	FFA	0.510	**
	C12:0	0.439	**	C14:0	0.377	*
	C14:0	0.441	**	C16:1	0.531	***
	C16:1	0.360	*	C18:2	0.441	**
	C18:1	0.429	**	C18:3 (n-3)	0.380	*
	C18:2	0.510	***			
	C20:4 (n-6)	0.372	*			
	C20:5 (n-3)	0.341	*			
**AT Oxylipids**	8-iso-PGF2 alpha	0.415	**	8-iso-PGF2 alpha	0.616	***
				15-HETE	0.631	***
				13-HODE	0.372	*
**AT gene expression**	*15LOX*	-0.519	**	*15LOX*	-0.692	***
	*ARG1*	-0.371	*	*ARG1*	-0.396	*
	*CYP2J2*	0.578	***	*CYP2J2*	0.385	*
	*CD44*	0.422	**	*CD68*	0.601	***
	*CD68*	0.635	***	*SPP1*	0.374	*
	*SPP1*	0.450	**			

HODE = hydroxyoctadecadienoic acid; HETE = hydroxyeicosatetraenoic acid.

* *P* < 0.05

** *P* < 0.01

*** *P* < 0.001

Reflective of enhanced lipolysis around parturition, AT content of C16:0 was reduced at PP compared to G1 and G2 (Table 1). Compared to G1, C16:1, LA and C18:3 (n-3) concentrations in AT were decreased at both G2 and PP. AT concentrations of C18:1 tended to decrease at G2 compared to G1 and PP (*P* = 0.1). ArA content in AT tended to decrease at G2 and PP compared to G1(*P* = 0.09). There were no differences in the AT content of FA measured between HL and LL.

A total of 18 ArA oxidized metabolites were analyzed in plasma and AT using our targeted lipidomic profile (Table 3). Plasma 5,6 lipoxin A4 and PGE_2_ were not detected in more than 50% of the samples and were not included in the analyses. The thresholds for limits of detection were calculated as described in [23]. Sampling time had no effect on the concentration of COX-derived plasma ArA oxylipids. The CyP450-derived 11,12-EET, 14,15-EET, 14,15-DHET, and 20-HETE increased at PP compared to G1 and G2. Similarly, circulating content of 8,9-EET tended to be higher at PP compared to other sampling time points (*P =* 0.06). Following a similar pattern to CYP450-derived EET, LOX metabolite 5-HETE increased after parturition. No differences in plasma ArA-derived oxylipids were detected between HL and LL.

**Table 3:** Arachidonic acid-derived oxylipid contents in plasma and adipose tissues from dairy cows are modified during the periparturient period. Adipose and plasma samples were collected at -27±7 (G1) and -10±5 d (G2) prepartum and at 8±3 d postpartum (PP). Cows were grouped by lipolysis intensity (LI) based on FFA concentrations at PP in low (LL = FFA<1.0 mEq/L) and high (HL, FFA≥1.0 mEq/L) lipolysis rate.

			G1		G2		PP			P-value		
	Biosynthesis Pathway	Arachidonic acid derived oxylipids^1^	LL	HL	LL	HL	LL	HL	SEM	Time	LI	Time by LI
**Plasma n***M*	**COX**^**3**^	6ketoPGF1alpha	0.363	0.610	0.454	0.263	0.790	0.440	0.385	ns^2^	ns	ns
		15dPGJ2	0.003	0.001	0.001	0.002	0.010	0.001	0.002	ns	ns	ns
		PGD2	0.086	0.229	0.642	0.225	0.115	0.161	0.189	ns	ns	ns
		TXB2	2.920	3.490	3.240	1.290	5.500	5.900	2.710	ns	ns	ns
	**CyP450**^**4**^	8,9-EET	0.033	0.035	0.041	0.029	0.144	0.487	0.131	0.06	ns	ns
		11,12-EET	0.036	0.010	0.010	0.044	0.119	0.105	0.027	<0.01	ns	ns
		14,15-EET	0.085	0.060	0.055	0.095	0.430	0.735	0.210	<0.05	ns	ns
		20-HETE	0.112	0.076	0.181	0.097	0.580	0.740	0.215	<0.01	ns	ns
		8,9-DHET	0.339	0.424	0.323	0.378	0.276	0.646	0.164	ns	ns	ns
		11,12-DHET	0.801	0.565	0.896	0.943	0.462	0.805	0.212	ns	ns	ns
		14,15-DHET	0.648	1.020	0.614	0.669	1.770	2.300	0.241	<0.05	ns	ns
	**LOX**^**5**^	5-HETE	0.781	0.398	0.265	0.581	2.000	6.140	0.985	<0.01	ns	ns
		5-oxoETE	0.051	0.070	0.022	0.020	0.107	0.122	0.252	ns	ns	ns
		15-HETE	0.055	0.056	0.025	0.068	0.214	0.147	0.092	ns	ns	ns
		15-oxoETE	0.010	0.017	0.005	0.010	0.038	0.016	0.011	ns	ns	ns
		11-HETE	0.071	0.057	0.022	0.067	0.138	0.139	0.053	ns	ns	ns
	**NE**^**6**^	8-iso-PGF2alpha	0.479	0.543	0.302	0.154	0.418	0.787	0.245	ns	ns	ns
		8,12-iso-iPF2alpha-VI	0.197	0.348	0.173	0.775	0.410	0.682	0.252	ns	ns	ns
**Adipose Tissue** μM/μg	**COX**	5,6-LipoxinA4	0.250	0.060	0.360	0.490	0.410	0.220	0.280	ns	ns	ns
		6ketoPGF1alpha	0.180	0.260	0.120	0.177	1.950	0.560	0.770	ns	ns	ns
		15dPGJ2	0.001	0.001	0.001	0.002	0.010	0.003	0.003	ns	ns	ns
		PGD2	0.130	0.070	0.010	0.010	0.220	0.310	0.170	0.09	ns	ns
		PGE2	0.070	0.130	0.230	0.090	0.720	1.350	0.500	0.06	ns	ns
		TXB2^7^	0.210	0.720	0.140	0.080	0.060	0.190	0.650	ns	ns	ns
	**CyP450**	8,9-EET	0.001	0.001	0.003	0.001	0.001	0.001	0.001	ns	ns	ns
		11,12-EET	0.001	0.002	0.002	0.001	0.001	0.001	0.000	ns	ns	ns
		14,15-EET	0.001	0.001	0.002	0.001	0.008	0.020	0.014	ns	ns	ns
		20-HETE	0.004	0.010	0.010	0.003	0.004	0.006	0.006	ns	ns	ns
		8,9-DHET^8^	0.003	0.001	0.003	0.003	0.007	0.009	0.004	ns	ns	ns
		11,12-DHET	0.008	0.032	0.020	0.010	0.010	0.007	0.012	ns	ns	ns
		14,15-DHET	0.020	0.020	0.010	0.020	0.180	0.090	0.070	ns	ns	ns
	**LOX**	5-HETE	0.220	0.130	0.690	0.430	0.750	0.470	0.180	<0.05	ns	ns
		5-oxoETE	0.001	0.001	0.010	0.003	0.010	0.007	0.004	ns	ns	ns
		15-HETE	0.050^b^^b^	0.030^a^	0.070^b^	0.040^a^	0.180^d^	0.090^c^	0.015	<0.05	<0.05	<0.05
		15-oxoETE	0.030	0.040	0.120	0.040	0.230	0.140	0.180	ns	ns	ns
	**NE**	11-HETE	0.070	0.120	0.110	0.060	0.290	0.190	0.040	<0.05	ns	ns
		8-iso-PGF2alpha	0.004	0.009	0.007	0.002	0.010	0.050	0.010	ns	ns	ns
		8,12-iso-iPF2alpha-VI	0.030	0.040	0.060	0.080	0.070	0.050	0.020	ns	ns	ns

^1^ Values are least squares means of arachidonic acid derived oxylipid concentrations in plasma and adipose tissue as measured in HPLC MS/MS.

^2^ ns = P > 0.10.

^3^ COX, cyclooxygenase

^4^ CyP450, cytochrome P450

^5^ LOX, lipoxygenase

^6^ NE, non-enzymatic oxidation

^7^ An outlier from HL treatment at G1 was removed for TXB2 data analysis

^8^ An outlier from HL treatment at G2 was removed for 8,9-DHET data analysis

^a-d^ least squares means with different letters differ (P<0.05)

In AT, PGD_2_ and PGE_2_ content tended to increase at PP compared to G1 and G2 (Table 3). LOX-derived 5-HETE increased at G2 and its concentration remained elevated at PP. The concentrations of 15-HETE in AT were higher at PP compared to G1. HL lowered AT concentrations of 15-HETE compared to LL at all sampling time points. AT content of the non-enzymatically derived 11-HETE was increased at PP compared to the prepartum sampling time points. Circulating FFA were associated with AT content of 8-iso-PGF2 alpha (Table 2). There was a positive association between adipose concentrations of 8-iso-PGF2 alpha and 15-HETE and circulating BHB (Table 2).

Of the 8 LA-derived oxylipids analyzed, the DiHOME and HODE families were the most abundant in plasma (Table 4). Circulating content of 9-HODE remained constant throughout the study and 13-HODE concentrations reached their lowest concentration at G2, but returned to G1 levels at PP. In contrast, plasma 9-oxoODE decreased at G2 and PP compared to G1. CyP450 derived 9,10-DiHOME and 12,13-DiHOME decreased in plasma after parturition. Plasma concentrations of 9,10-EpOME were lower at G2 compared to G1. Similarly, circulating content of 12,13-EpOME was reduced at G2 compared to G1 and PP.

**Table 4:** Linoleic acid derived oxylipid contents in plasma and adipose tissues from dairy cows are modified during the periparturient period. Adipose and plasma samples were collected at -27±7 (G1) and -10±5 d (G2) prepartum and at 8±3 d postpartum (PP). Cows were grouped by lipolysis intensity (LI) based on FFA concentrations at PP in low (LL = FFA<1.0 mEq/L) and high (HL, FFA≥1.0 mEq/L) lipolysis rate.

			G1		G2		PP			P-value		
	Biosynthesis Pathway	Linoleic acid derived oxylipids^1^	LL	HL	LL	HL	LL	HL	SEM	Time	LI	Time by LI
**Plasma n***M*	**LOX/NE**^**3**^	9-HODE	9.37	3.91	7.21	3.15	14.30	9.87	7.76	ns^2^	ns	ns
		9-oxoODE	6.64	5.42	3.67	1.96	1.87	3.17	1.75	<0.05	ns	ns
		13-HODE	24.50	30.02	16.46	11.22	29.33	22.11	4.67	<0.05	ns	ns
		13-oxoODE	2.94	1.84	0.75	1.21	2.56	3.49	1.73	ns	ns	ns
	**CyP450**^**4**^	9,10-EpOME	0.89	0.44	0.17	0.31	0.37	0.95	0.12	<0.05	ns	ns
		9,10-DiHOME	4.01	4.25	4.63	3.78	2.89	1.41	0.54	<0.05	ns	ns
		12,13-EpOME	5.24	5.05	1.87	2.61	5.05	6.86	2.14	<0.05	ns	ns
		12,13-DiHOME	13.95	8.06	8.36	9.50	5.86	4.46	3.60	<0.05	ns	ns
**Adipose Tissue** μM/μg	**LOX/NE**	9-HODE	7.10	4.10	10.00	3.10	24.70	14.90	7.20	0.10	ns	ns
		9-oxoODE	4.90	4.70	13.60	7.10	10.30	6.80	3.50	ns	ns	ns
		13-HODE	7.90	5.80	6.90	5.10	13.32	12.52	1.06	0.05	ns	ns
		13-oxoODE	3.50	4.90	6.90	5.33	8.40	12.80	5.30	ns	ns	ns
	**CyP450**	9,10-EpOME	0.10	0.24	0.16	0.28	0.20	0.49	0.22	ns	ns	ns
		9,10-DiHOME	3.13	2.83	2.43	2.35	1.82	1.21	1.23	<0.05	ns	ns
		12,13-EpOME	1.40	1.10	3.20	1.30	2.20	1.46	0.62	ns	ns	ns
		12,13-DiHOME	0.52	0.41	0.27	0.33	0.40	0.52	0.06	<0.05	ns	ns

^1^ Values are least squares means of linoleic acid derived oxylipid concentrations in plasma and adipose tissue as measured in HPLC MS/MS.

^2^ ns = P > 0.10.

^3^ LOX/NE, lipoxygenase/ non-enzymatic oxidation

^4^ CyP450, cytochrome P450

In AT, 9-HODE tended to increase at PP in comparison to G1 and G2 (Table 4). In the same way, AT 13-HODE increased after parturition (Table 4). The content of 9,10-DiHOME in AT was reduced at PP compared to G1. The AT content of 12,13-DiHOME was reduced at G2 compared to G1 and PP. Partial correlation analysis demonstrated a positive association between 13-HODE content in AT and circulating BHB (Table 2).

### AT expression of oxylipid enzymes and inflammatory markers

Gene expression of enzymes that oxidize PUFA was analyzed in AT samples by RT-qPCR (Table 5). After parturition, the expression of *ALOX15* was progressively reduced as the periparturient period progressed. Similarly, the transcription of *EPHX2* (encoding sEH) was reduced at PP compared to G1. In contrast, the transcription of *ALOX5* increased at PP compared to G1 and G2 and was higher in HL. *CYP2J2* expression was upregulated after parturition. The gene expression of *PTGS2* and *CYP2A4* remained unchanged throughout the periparturient period. Partial correlation analyses indicated that circulating concentrations of FFA and BHB were negatively associated with the gene expression of *ALOX15* (Table 2). In contrast, the expression of *CYP2J2* was positively correlated with circulating FFA and BHB (Table 2).

**Table 5:** Gene expression of oxylipid enzymes, inflammatory cytokines, and macrophage markers in adipose tissue is affected by lipolysis intensity and time relative to parturition. Relative abundance of mRNA in subcutaneous adipose tissue collected at -27±7 (G1) and -10±5 d (G2) prepartum and at 8±3 d postpartum (PP). Cows were grouped by lipolysis intensity (LI) based on FFA concentrations at PP in low (LL = FFA<1.0 mEq/L) and high (HL, FFA≥1.0 mEq/L) lipolysis rate.

		G1		G2		PP			P-value		
Gene Network	Gene^1^	LL	HL	LL	HL	LL	HL	SEM	Time	LI	Time by LI
**Oxylipid enzymes**	*ALOX5*	0.54	1.17	0.83	1.70	1.24	1.56	0.26	<0.05	<0.01	ns^2^
	*ALOX15*	2.84	4.01	0.93	2.32	0.45	0.45	0.43	<0.001	ns	ns
	*CYP2A4*	1.23	1.15	0.74	1.31	2.47	2.41	0.80	ns	ns	ns
	*CYP2J2*	0.64	1.40	0.75	1.79	2.27	4.68	0.80	<0.05	ns	ns
	*PTGS2*	1.34	0.94	1.87	0.85	1.55	1.11	0.55	ns	ns	ns
** **	*EPHX2*	1.25	1.29	1.25	1.51	0.56	0.71	0.17	<0.05	ns	ns
**Inflammatory cytokines, macrophage infiltration markers**	*TNF*	1.00	1.95	0.79	1.07	1.38	1.33	0.35	ns	ns	ns
	*IL6*	1.25	0.90	0.73	1.19	1.38	1.36	0.37	ns	ns	ns
	*IL10*	0.48	1.26	0.58	1.28	4.17	2.69	0.98	<0.01	ns	ns
	*CCL2*	0.65	2.17	1.58	1.75	0.57	1.53	0.51	ns	ns	ns
	*ARG1*	3.06	4.12	1.88	3.11	0.34	0.39	0.92	<0.05	ns	ns
	*CCL22*	0.93	1.66	1.63	6.64	0.34	3.14	1.45	ns	ns	ns
	*CD68*	0.60	1.34	0.48	1.17	1.60	4.90	0.68	<0.01	<0.05	ns
	*CD44*	0.83	1.26	0.92	1.14	1.10	1.54	0.20	ns	ns	ns
	*SPP1*	0.38	7.43	0.14	3.91	1.66	13.29	3.99	<0.05	<0.01	ns

^1^ Values are least squares means of the relative expression of genes *ALOX5* (encoding: 5-lipoxygenase), *ALOX15* (15-lipoxygenase), *CYP2A4* (cytochrome P450 2A4), *CYP2J2* (cytochrome P450 2J2), *PTGS2* (cyclooxygenase 2), *EPHX2* (soluble epoxy hydrolase0, *TNF* (tumor necrosis alpha), *IL6*, *IL10* (interleukins 6 and 10), *CCL2* (MCP-1), *ARG1* (arginase 1), *CCL22* (C-C motif chemokine ligand 22), CD68 (cd68), *CD44* (cd44), *SPP1* (osteopontin)

^2^ ns = P > 0.10.

The expression of gene markers of inflammation and macrophage infiltration in AT was also evaluated (Table 5). No changes in the gene transcription of pro-inflammatory cytokines *TNFa* (encoding tumor necrosis factor alpha), *IL6* (encoding interleukin 6), *CCL2* (encoding macrophage chemoattractant protein 1), or the anti-inflammatory cytokine *CCL22* (encoding C-C motif chemokine 22), were observed. In contrast, the expression of macrophage marker *CD68* and the gene encoding the macrophage chemoattractant osteopontin *SPP1*, [26] were upregulated at PP compared to G1 and G2. The expression of both genes was elevated across time in HL compared to LL (Table 5). The expression of the anti-inflammatory cytokine IL10 was upregulated after parturition. In contrast, the transcription of the gene encoding arginase 1 (*ARG1*), a marker of anti-inflammatory macrophages, was downregulated after calving. The expressions of *CD68* and *SPP1* in AT were positively associated with circulating BHB and FFA (Table 2). *CD44* transcription was similarly correlated with FFA in plasma. In contrast, *ARG1* transcription was negatively associated with BHB and FFA (Table 2).

## Discussion

Hormonal changes associated with parturition and lactation, along with reduced energy intake, promote lipolysis and increase circulating FFA in the first weeks after calving (reviewed in [7]). Although lipolysis intensity may be further affected by changes in neural, hepatic, and pancreatic functions this study focused on changes within AT that may enhance its responses to lipolytic stimuli. In the present study, increased lipolysis intensity (HL) was associated with HSL activity (assessed as serine 660 phosphorylation) which is consistent with previous reports that highlight the role of HSL over other lipases in the lipolytic response following parturition [27, 28]. Lipolysis intensity in the present group of periparturient cows was dependent on adipocyte area prior to parturition and HL cows exhibited a greater reduction in adipocyte area after parturition. Similar results were reported by De Koster et al., [29] who showed an association between adipocyte size and lipolysis intensity. Data from our present study and others indicate that the magnitude of the postpartum lipolytic response is dependent of adipocyte size in the early dry period.

We previously reported the effect of periparturient lipolysis on the composition of total lipid FA profiles in plasma [30]. In the present study, we focused on specific FA, that have been associated with dynamic changes in plasma and AT during the periparturient period and that modify or are substrates for oxylipid biosynthesis [8, 30]. During lipolysis, FA are released from triglycerides stored in the lipid droplet of adipocytes through sequential hydrolysis by adipose triglyceride lipase, HSL, and monoglyceride lipase [31]. HSL activity is dependent on FA and positional specificity, preferring FA with chains of 20 carbon atoms or less and those located at the *sn-1* or *sn-3* positions of the triglyceride molecule [32, 33]. For a given chain length, FA hydrolysis increases with increased unsaturation [33]. In our present study, HSL specificity was reflected in the FA profile of plasma at PP with increased content of saturated and unsaturated FA with chains of 20 or less carbons. For 16 and 18 carbons FA, we observed that the degree of unsaturation enhanced the release of FA from AT into circulation. For example, the plasma concentration of stearic acid (C18:0) did not change throughout the present study, while all the unsaturated 18 carbon FA measured increased. Minimal mobilization of stearic acid during the periparturient period of dairy cows was also reported previously [8, 30]. Our observation that C18:0, ArA, C20:5(n-3), and C22:6n3 were more abundant in the plasma of LL compared to HL may indicate that the release rate of these long chain FA is related to the size of the lipid droplet (i.e. adipocyte area) and the abundance of preferentially mobilized saturated FA in HL. Our results on FA mobilization dynamics support a relative specificity in the release of saturated FA during lipolysis in periparturient dairy cows. This finding suggests that carbon chain length and the degree of unsaturation may be utilized for targeted supplementation with the goal of modifying FFA profiles of periparturient dairy cows. Since, increasing the availability of certain long chain PUFA (e.g. alpha-linolenic, EPA, and DHA) in plasma of periparturient cows was shown to modulate inflammatory responses around parturition [9, 34], further studies on FA trafficking in AT and plasma are needed to improve the efficacy of lipid supplementation in the dry and periparturient periods.

ArA-derived oxylipid profiles differed between AT and plasma of all cows in the present study. This observation confirms that oxylipid profiles appear to be organ or tissue specific and changes in their content are not always reflected in blood. For example, in dairy cows with clinical coliform mastitis and their healthy matching controls, the oxylipid composition of milk was shown to differ from that of plasma [23]. More recently, Kuhn et al., [16] demonstrated that differences between milk and plasma oxylipid profile are maintained during different stages of lactation. Differences in oxylipid profiles among plasma and peripheral organs are also described in other species. For example, in a rodent model of sepsis, AT eicosanoid profile was not reflected in plasma of both LPS-treated and control animals [35]. These findings suggest that plasma oxylipid concentration and composition do not reflect those of the adipose organ, at least during the periparturient period.

Plasma concentrations of ArA metabolites from the COX pathway are reported to remain unchanged throughout the periparturient period in healthy cows [15], and our results confirmed the lack of variation in these circulating oxylipids. In contrast, the CYP450 product 20-HETE was increased in plasma from all cows at PP in the present study. This observation coincides with results from Kuhn et al., [16] where 20-HETE was increased in plasma from cows at 1–2 days after parturition compared to cows at 80–95 and 184–207 days into lactation. The source of the postpartum peak of plasma 20-HETE observed in our study is unclear, but it may be related to post-calving uterine involution. In periparturient rodents, 20-HETE promotes uterine contractility and size reduction by promoting vasoconstriction of myometrial arteries [36]. The physiological relevance of postpartum 20-HETE in periparturient dairy cows remains to be elucidated.

A second ArA metabolite that exhibited interesting dynamics during the periparturient period was PGE_2_. Lipolysis in 3T3-L1 adipocytes increases the concentration of PGE_2_ [11, 37], which in turn, promotes the secretion of macrophage chemoattractant protein 1, a potent chemotactic peptide that drives macrophage infiltration into tissues [37]. A clinical study of obese humans with elevated omental AT lipolysis rate also described increased concentrations of PGE_2_ when compared to healthy volunteers [38]. In agreement with these reports, cows in our experiment showed a tendency for increased AT PGE_2_ at PP. The impact of enhanced PGE_2_ release during lipolysis is unclear. Some studies report beneficial effects that appear to palliate chronic AT inflammation in obesity [38], while others indicate that reducing PGE_2_ secretion through nonsteroidal anti-inflammatory drugs minimizes AT macrophage infiltration [39]. Although conjecture, in dairy cows, PGE_2_ may have potent anti-lipolytic activity similar to other species [40]. This effect was indirectly suggested in a study by Bradford et al. [41] in which PGE_2_ concentrations were not measured, but anti-inflammatory salicylate treatment increased plasma FFA and enhanced milk fat secretion during early lactation. Revealing the role of COX-derived PGE_2_ and other prostaglandins in lipolysis modulation during early lactation may provide nutritional or pharmacological targets for minimizing periparturient lipolysis.

The biosynthesis of ArA metabolites derived from LOX is commonly associated with dysregulation of AT function. For example, in obese mice, AT secretion of 5-HETE and 15-HETE is increased as the capacity of adipocytes to respond to the anti-lipolytic action of insulin is decreased [42]. The main sources of these LOX metabolites are macrophages and adipocytes that secrete them in response to exposure to C16:0 and LA [42, 43]. The increased content of 5-HETE in AT and plasma of cows at PP in the present study may be explained in part by the increased availability of both C16:0 and LA during PP because both FA promote the biosynthesis of 5-HETE by AT macrophages [42]. Given the angiogenic and adipogenic effects of 5-HETE [44, 45], this metabolite may promote adequate AT remodeling during intense lipolysis, as both processes are necessary for inflammation resolution [4]. A third ArA metabolite that fluctuated in AT during the present study was 15-HETE. This LOX-derived oxylipid was reduced in AT from HL compared to LL. Adipocytes are an important source of 15-HETE and, once secreted, it promotes adipogenesis and lipogenesis by enhancing the activation of protein kinase B and acting as a PPARγ agonist [46]. The impact of reduced levels of 15-HETE in HL is unclear, although PPARγ activation during the periparturient period is necessary to modulate lipolysis by promoting adipogenic responses and minimizing FFA release [47]. Although there are several PPARγ agonists that differ in affinity for and effects on the receptor [48], the potency of 15-HETE as a PPARγ ligand and its effects on AT function in dairy cows warrants further investigation.

This is the first study to our knowledge that reports the dynamics of LA-derived oxylipids in AT of periparturient dairy cows. CYP450-derived vicinal diols (i.e. hydroxyl groups are attached to adjacent carbons), including 9,10- and 12,13-DiHOME, were abundant in both AT and plasma. These metabolites are the product of serial oxygenations and hydrations by CYP450 and soluble epoxide hydrolase, respectively [49]. The role of 9,10-DiHOME in AT function is not completely defined, but this metabolite is characterized, together with 12,13-DiHOME, as an inhibitor of mitochondrial function and promotor of oxidative stress [50]. In rodents, 12,13-DiHOME is adipogenic and promotes FA uptake in adipocytes [51]. Given the adipogenic effects of these LA diols and their observed reduction in plasma and AT after parturition, a better characterization of their effects on bovine adipocytes is warranted. Enhancing lipogenic activity in AT is beneficial for dairy cows during early lactation as this would translate into a net reduction in the release of FFA into circulation and increased dry matter intake [52].

The dynamics of plasma 9-HODE observed in our study are similar to those described by Raphael et al., [15] that followed dairy cows through the periparturient period and into mid-lactation. In that study, 9-HODE remained constant around calving and only peaked at 85 d after parturition. It is important to note that 9-HODE may increase at parturition, as reported by Bradford et al., [53], however, our sampling protocol and that of Raphael et al. (2014), did not include this time point. Plasma 13-HODE was reduced prior to parturition in the present study and in the report by Raphael et al. [15]. Remarkably, AT content of 13-HODE contrasted with its plasma concentrations as it increased at a steady rate during the periparturient period. 13-HODE is a potent PPARγ agonist that promotes adipogenesis [54]. During lipolysis, this LA metabolite acts as a chemoattractant for macrophages by enhancing migration into AT [12]. Excessive accumulation of 9- and 13-HODE in tissues leads to chronic inflammation that is characterized by macrophage infiltration and formation of foam cells in the case of atherosclerosis lesions and crown-like structure cells in the case of obesity [12, 43]. In our group of cows, the peak in AT content of 13-HODE coincided with a higher gene expression of the macrophage specific marker *CD68* and the macrophage chemoattractant osteopontin (*SPP1*) [26]. Thus the role of HODE in AT inflammation during the periparturient period of dairy cows warrants a thorough characterization, especially since accumulation of AT macrophages is a common finding in cows with metabolic diseases in early lactation [5].

Depending on the PUFA substrate, oxylipids can be independently or collectively synthesized by different enzymatic and non-enzymatic pathways. We evaluated the gene expression of a select group of oxylipid enzymes, however, given the substrate redundancy of some of these enzymes and that some products (e.g. 9- and 13-HODE) can also be produced by non-enzymatic reactions, the expression activity may not always be associated with the abundance of a specific metabolite. In the present study, we observed an increase in the expression of *CYP2J2* at PP. This gene encoding an isoform of CYP450 is abundantly expressed in AT, where it also hydroxylases vitamin D [55]. Remarkably, in obese women, the expression of this gene is reduced and explains, in part, the limited availability of 1,25-vitamin D in these subjects [55]. It is uncertain if the enhanced expression of *CYP2J2* observed in this group of periparturient dairy cows is indicative of a more prominent role of AT in the metabolism of vitamin D. Therefore, the implications of *CYP2J2* activity in AT for adequate metabolism of vitamin D in periparturient cows remain to be established.

In contrast to studies of the bovine mammary gland in which *ALOX15* expression is shown to increase dramatically after parturition [56], the transcription of this gene was reduced in AT at G2 and PP. The activity of this enzyme, and more specifically its metabolites 9- and 13-HODE, was recently linked to enhanced macrophage trafficking in AT during lipolysis in mice [54]. Since the enzymatic activity of COX and CYP450 can also yield 9- and 13-HODE from LA [57, 58], the increased concentrations of these metabolites in AT of cows in our present study may be independent of the reduced expression of *ALOX15* in AT. The expression of *EPHX2* was also downregulated at PP. This gene encodes the enzyme soluble epoxide hydrolase and its degree of expression is reflected in protein content of AT [59]. This enzyme acts on CYP450-derived epoxides to yield FA diols. During the differentiation of 3T3-L1 cells, the gene and protein expression of soluble epoxide hydrolase is increased while those of CYP2J isoforms are decreased [59]. In the present study, the *EPHX2* expression pattern in AT matched the plasma and AT content of its metabolites, with both 9,10- and 12, 13-DiHOME being reduced around parturition. In rodents, limiting activity of soluble epoxide hydrolase by pharmacological suppression with the specific inhibitor AUDA decreases plasma content of 9,10- and 12,13-DiHOME [60]. The physiological effect of limited AT content of these DiHOME around parturition is unknown; however, their capacity to promote FA uptake by adipocytes [51] may be beneficial for periparturient dairy cows that have limited capacity for buffering excess FA during the periparturient period.

## Conclusion

Although AT function during the periparturient period is modulated by hepatic, pancreatic, and central nervous system activity, results from this study highlight that lipolysis products should be considered as possible mechanisms regulating the adaptation of AT to increased energy demands around parturition. As in rodents and humans [2, 61], the activation of lipolytic pathways could induce inflammatory responses and changes in AT immune cell trafficking. These alterations in inflammation patterns may be modulated in part by oxylipids generated during lipolysis [11]. Results from our study indicate that changes in the oxylipid profiles of AT and plasma are linked with lipolysis intensity. Enhanced availability of LA coincided with increased 13-HODE content in AT and plasma. At the same time, lipolysis was associated with increased AT concentrations of ArA metabolites including 15-HETE. Elucidating the effects of these ArA- and LA-derived oxylipids on AT function during the periparturient period will aid the development of nutritional interventions that can modulate the deleterious effects of excessive lipolysis on dairy cow health and lactation performance.

## Supporting information

S1 File Supplementary Tables A, B, C, and D.
(DOCX)

## References

[1] Bradford BJ, Yuan K, Farney JK, Mamedova LK, Carpenter AJ. Invited review: Inflammation during the transition to lactation: New adventures with an old flame. J Dairy Sci. 2015;98(10):6631–50. Epub 2015/07/27. 10.3168/jds.2015-9683 . 26210279

[2] Kosteli A, Sugaru E, Haemmerle G, Martin JF, Lei J, Zechner R, et al Weight loss and lipolysis promote a dynamic immune response in murine adipose tissue. J Clin Invest. 2010;120(10):3466–79. 10.1172/JCI42845 20877011 PMC2947229

[3] Contreras GA, Strieder-Barboza C, Raphael W. Adipose tissue lipolysis and remodeling during the transition period of dairy cows. J Anim Sci Biotechnol. 2017;8(1):41 10.1186/s40104-017-0174-4 28484594 PMC5420123

[4] Lee Y-H, Mottillo EP, Granneman JG. Adipose tissue plasticity from WAT to BAT and in between. Biochim Biophys Acta. 2014;1842(3):358–69. 10.1016/j.bbadis.2013.05.011 23688783 PMC4435780

[5] Contreras GA, Kabara E, Brester J, Neuder L, Kiupel M. Macrophage infiltration in the omental and subcutaneous adipose tissues of dairy cows with displaced abomasum. J Dairy Sci. 2015;98(9):6176–87. 10.3168/jds.2015-9370 26117355

[6] Sordillo LM, Raphael W. Significance of Metabolic Stress, Lipid Mobilization, and Inflammation on Transition Cow Disorders. Vet Clin North Am Food Anim Pract. 2013;29(2):267–78. 10.1016/j.cvfa.2013.03.002 23809891

[7] McArt JAA, Nydam DV, Oetzel GR, Overton TR, Ospina PA. Elevated non-esterified fatty acids and β-hydroxybutyrate and their association with transition dairy cow performance. The Veterinary Journal. 2013;198(3):560–70. 10.1016/j.tvjl.2013.08.011 24054909

[8] Douglas GN, Rehage J, Beaulieu AD, Bahaa AO, Drackley JK. Prepartum nutrition alters fatty acid composition in plasma, adipose tissue, and liver lipids of periparturient dairy cows. J Dairy Sci. 2007;90(6):2941–59. Epub 2007/05/23. 10.3168/jds.2006-225 . 17517735

[9] Zachut M, Arieli A, Lehrer H, Livshitz L, Yakoby S, Moallem U. Effects of increased supplementation of n-3 fatty acids to transition dairy cows on performance and fatty acid profile in plasma, adipose tissue, and milk fat. J Dairy Sci. 2010;93(12):5877–89. Epub 2010/11/26. 10.3168/jds.2010-3427 . 21094761

[10] Barquissau V, Ghandour RA, Ailhaud G, Klingenspor M, Langin D, Amri E-Z, et al Control of adipogenesis by oxylipins, GPCRs and PPARs. Biochimie. 2017;136:3–11. 10.1016/j.biochi.2016.12.012 28034718

[11] Gartung A, Zhao J, Chen S, Mottillo E, VanHecke GC, Ahn YH, et al Characterization of Eicosanoids Produced by Adipocyte Lipolysis: IMPLICATION OF CYCLOOXYGENASE-2 IN ADIPOSE INFLAMMATION. J Biol Chem. 2016;291(31):16001–10. Epub 2016/06/02. 10.1074/jbc.M116.725937 27246851 PMC4965551

[12] Lee YH, Kim SN, Kwon HJ, Maddipati KR, Granneman JG. Adipogenic role of alternatively activated macrophages in beta-adrenergic remodeling of white adipose tissue. Am J Physiol Regul Integr Comp Physiol. 2016;310(1):R55–65. Epub 2015/11/06. 10.1152/ajpregu.00355.2015 26538237 PMC4796635

[13] Sodhi K, Puri N, Inoue K, Falck JR, Schwartzman ML, Abraham NG. EET agonist prevents adiposity and vascular dysfunction in rats fed a high fat diet via a decrease in Bach 1 and an increase in HO-1 levels. Prostaglandins & Other Lipid Mediators. 2012;98(3–4):133–42. 10.1016/j.prostaglandins.2011.12.004 22209722 PMC3449325

[14] López-Vicario C, Alcaraz-Quiles J, García-Alonso V, Rius B, Hwang SH, Titos E, et al Inhibition of soluble epoxide hydrolase modulates inflammation and autophagy in obese adipose tissue and liver: Role for omega-3 epoxides. Proceedings of the National Academy of Sciences. 2015;112(2):536–41. 10.1073/pnas.1422590112 25550510 PMC4299190

[15] Raphael W, Halbert L, Contreras GA, Sordillo LM. Association between polyunsaturated fatty acid-derived oxylipid biosynthesis and leukocyte inflammatory marker expression in periparturient dairy cows. J Dairy Sci. 2014;97(6):3615–25. Epub 2014/04/16. 10.3168/jds.2013-7656 . 24731638

[16] Kuhn MJ, Mavangira V, Gandy JC, Zhang C, Jones AD, Sordillo LM. Differences in the Oxylipid Profiles of Bovine Milk and Plasma at Different Stages of Lactation. Journal of Agricultural and Food Chemistry. 2017;65(24):4980–8. 10.1021/acs.jafc.7b01602 28570057

[17] Strieder-Barboza C, Zondlak A, Kayitsinga J, Pires AFA, Contreras GA. Lipid mobilization assessment in transition dairy cattle using ultrasound image biomarkers. Livestock Science. 2015;177:159–64. 10.1016/j.livsci.2015.04.020 WOS:000356987900020.

[18] Kabara E, Sordillo LM, Holcombe S, Contreras GA. Adiponectin links adipose tissue function and monocyte inflammatory responses during bovine metabolic stress. Comp Immunol Microbiol Infect Dis. 2014;37(1):49–58. 10.1016/j.cimid.2013.10.007 24296305

[19] Vandesompele J, De Preter K, Pattyn F, Poppe B, Van Roy N, De Paepe A, et al Accurate normalization of real-time quantitative RT-PCR data by geometric averaging of multiple internal control genes. Genome Biology. 2002;3(7):research0034.1–research.11. PMC126239. 12184808 10.1186/gb-2002-3-7-research0034 PMC126239

[20] Hellemans J, Mortier G, De Paepe A, Speleman F, Vandesompele J. qBase relative quantification framework and software for management and automated analysis of real-time quantitative PCR data. Genome Biology. 2007;8(2):R19–R. 10.1186/gb-2007-8-2-r19 PMC1852402. 17291332 PMC1852402

[21] Contreras GA, Thelen K, Schmidt SE, Strieder-Barboza C, Preseault CL, Raphael W, et al Adipose tissue remodeling in late-lactation dairy cows during feed-restriction-induced negative energy balance. J Dairy Sci. 2016;99(12):10009–21. 10.3168/jds.2016-11552 27720147

[22] Contreras GA, Thelen K, Ayala-Lopez N, Watts SW. The distribution and adipogenic potential of perivascular adipose tissue adipocyte progenitors is dependent on sexual dimorphism and vessel location. Physiological Reports. 2016;4(19). doi: ARTN e12993 10.14814/phy2.12993 WOS:000387445200005. 27738018 PMC5064145

[23] Mavangira V, Gandy JC, Zhang C, Ryman VE, Daniel Jones A, Sordillo LM. Polyunsaturated fatty acids influence differential biosynthesis of oxylipids and other lipid mediators during bovine coliform mastitis. J Dairy Sci. 2015;98(9):6202–15. Epub 2015/07/15. 10.3168/jds.2015-9570 . 26162796

[24] O'Donnell VB, Maskrey B, Taylor GW. Eicosanoids: generation and detection in mammalian cells. Methods Mol Biol. 2009;462:5–23. Epub 2009/01/24. . 19160658

[25] Ryman VE, Pighetti GM, Lippolis JD, Gandy JC, Applegate CM, Sordillo LM. Quantification of bovine oxylipids during intramammary Streptococcus uberis infection. Prostaglandins Other Lipid Mediat. 2015;121(Pt B):207–17. Epub 2015/10/04. 10.1016/j.prostaglandins.2015.09.006 . 26432060

[26] Lee Y-H, Petkova A, Granneman J. Identification of an adipogenic niche for adipose tissue remodeling and restoration. Cell Metab. 2013;18 10.1016/j.cmet.2013.08.003 24011071 PMC4185305

[27] Koltes DA, Spurlock DM. Coordination of lipid droplet-associated proteins during the transition period of Holstein dairy cows. J Dairy Sci. 2011;94(4):1839–48. 10.3168/jds.2010-3769 21426973

[28] Locher LF, Meyer N, Weber EM, Rehage J, Meyer U, Danicke S, et al Hormone-sensitive lipase protein expression and extent of phosphorylation in subcutaneous and retroperitoneal adipose tissues in the periparturient dairy cow. J Dairy Sci. 2011;94(9):4514–23. Epub 2011/08/23. 10.3168/jds.2011-4145 . 21854923

[29] De Koster J, Van den Broeck W, Hulpio L, Claeys E, Van Eetvelde M, Hermans K, et al Influence of adipocyte size and adipose depot on the in vitro lipolytic activity and insulin sensitivity of adipose tissue in dairy cows at the end of the dry period. J Dairy Sci. 2016;99(3):2319–28. Epub 2016/01/03. 10.3168/jds.2015-10440 . 26723122

[30] Contreras GA, O'Boyle NJ, Herdt TH, Sordillo LM. Lipomobilization in periparturient dairy cows influences the composition of plasma nonesterified fatty acids and leukocyte phospholipid fatty acids. J Dairy Sci. 2010;93(6):2508–16. Epub 2010/05/25. 10.3168/jds.2009-2876 . 20494158

[31] Frühbeck G, Méndez-Giménez L, Fernández-Formoso J-A, Fernández S, Rodríguez A. Regulation of adipocyte lipolysis. Nutrition Research Reviews. 2014;27(1):63–93. Epub 05/28. 10.1017/S095442241400002X 24872083

[32] Raclot T, Groscolas R. Differential mobilization of white adipose tissue fatty acids according to chain length, unsaturation, and positional isomerism. Journal of Lipid Research. 1993;34(9):1515–26. 8228635

[33] Raclot T, Holm C, Langin D. Fatty acid specificity of hormone-sensitive lipase: implication in the selective hydrolysis of triacylglycerols. Journal of Lipid Research. 2001;42(12):2049–57. 11734578

[34] Gandra JR, Barletta RV, Mingoti RD, Verdurico LC, Freitas JE Jr., Oliveira LJ, et al Effects of whole flaxseed, raw soybeans, and calcium salts of fatty acids on measures of cellular immune function of transition dairy cows. J Dairy Sci. 2016;99(6):4590–606. Epub 2016/04/12. 10.3168/jds.2015-9974 . 27060809

[35] Balvers MGJ, Verhoeckx KCM, Meijerink J, Bijlsma S, Rubingh CM, Wortelboer HM, et al Time-dependent effect of in vivo inflammation on eicosanoid and endocannabinoid levels in plasma, liver, ileum and adipose tissue in C57BL/6 mice fed a fish-oil diet. International Immunopharmacology. 2012;13(2):204–14. 10.1016/j.intimp.2012.03.022 22498761

[36] Pearson T, Warren AY, Barrett DA, Khan RN. Detection of EETs and HETE-generating cytochrome P450 enzymes and the effects of their metabolites on myometrial and vascular function. American Journal of Physiology—Endocrinology And Metabolism. 2009;297(3):E647–E56. 10.1152/ajpendo.00227.2009 19549792

[37] Hu X, Cifarelli V, Sun S, Kuda O, Abumrad NA, Su X. Major role of adipocyte prostaglandin E2 in lipolysis-induced macrophage recruitment. Journal of Lipid Research. 2016;57(4):663–73. 10.1194/jlr.M066530 26912395 PMC4808775

[38] García-Alonso V, Titos E, Alcaraz-Quiles J, Rius B, Lopategi A, López-Vicario C, et al Prostaglandin E2 Exerts Multiple Regulatory Actions on Human Obese Adipose Tissue Remodeling, Inflammation, Adaptive Thermogenesis and Lipolysis. PLoS ONE. 2016;11(4):e0153751 10.1371/journal.pone.0153751 27124181 PMC4849638

[39] Iyer A, Lim J, Poudyal H, Reid RC, Suen JY, Webster J, et al An Inhibitor of Phospholipase A Group IIA Modulates Adipocyte Signaling and Protects Against Diet-Induced Metabolic Syndrome in Rats. Diabetes. 2012;61(9):2320–9. 10.2337/db11-1179 22923652 PMC3425408

[40] Johansson SM, Yang JN, Lindgren E, Fredholm BB. Eliminating the antilipolytic adenosine A1 receptor does not lead to compensatory changes in the antilipolytic actions of PGE2 and nicotinic acid. Acta Physiologica. 2007;190(1):87–96. 10.1111/j.1365-201X.2007.01692.x 17428236

[41] Farney JK, Mamedova LK, Coetzee JF, KuKanich B, Sordillo LM, Stoakes SK, et al Anti-inflammatory salicylate treatment alters the metabolic adaptations to lactation in dairy cattle. American Journal of Physiology—Regulatory, Integrative and Comparative Physiology. 2013;305(2):R110–R7. 10.1152/ajpregu.00152.2013 23678026 PMC3727001

[42] Long EK, Hellberg K, Foncea R, Hertzel AV, Suttles J, Bernlohr DA. Fatty acids induce leukotriene C4 synthesis in macrophages in a fatty acid binding protein-dependent manner. Biochimica et Biophysica Acta (BBA)—Molecular and Cell Biology of Lipids. 2013;1831(7):1199–207. 10.1016/j.bbalip.2013.04.004 24046860

[43] Vangaveti V, Baune BT, Kennedy RL. Hydroxyoctadecadienoic acids: novel regulators of macrophage differentiation and atherogenesis. Ther Adv Endocrinol Metab. 2010;1(2):51–60. Epub 2010/04/01. 10.1177/2042018810375656 23148150 PMC3475286

[44] Chatterjee M, Das S, Roy K, Chatterjee M. Overexpression of 5-lipoxygenase and its relation with cell proliferation and angiogenesis in 7,12-dimethylbenz(α)anthracene-induced rat mammary carcinogenesis. Molecular Carcinogenesis. 2013;52(5):359–69. 10.1002/mc.21858 22213124

[45] Casado-Díaz A, Ferreiro-Vera C, Priego-Capote F, Dorado G, Luque-de-Castro MD, Quesada-Gómez JM. Effects of arachidonic acid on the concentration of hydroxyeicosatetraenoic acids in culture media of mesenchymal stromal cells differentiating into adipocytes or osteoblasts. Genes & Nutrition. 2013;9(1):375 10.1007/s12263-013-0375-1 24338342 PMC3896622

[46] Song Y-S, Lee DH, Yu J-H, Oh D-K, Hong JT, Yoon D-Y. Promotion of adipogenesis by 15-(S)-hydroxyeicosatetraenoic acid. Prostaglandins & Other Lipid Mediators. 2016;123:1–8. 10.1016/j.prostaglandins.2016.02.001 26905195

[47] Bionaz M, Chen S, Khan MJ, Loor JJ. Functional Role of PPARs in Ruminants: Potential Targets for Fine-Tuning Metabolism during Growth and Lactation. PPAR Research. 2013;2013:28 10.1155/2013/684159 23737762 PMC3657398

[48] Ahmadian M, Suh JM, Hah N, Liddle C, Atkins AR, Downes M, et al PPAR[gamma] signaling and metabolism: the good, the bad and the future. Nat Med. 2013;99(5):557–66. 10.1038/nm.3159 PMC3870016 23652116

[49] Konkel A, Schunck W-H. Role of cytochrome P450 enzymes in the bioactivation of polyunsaturated fatty acids. Biochimica et Biophysica Acta (BBA)—Proteins and Proteomics. 2011;1814(1):210–22. 10.1016/j.bbapap.2010.09.009 20869469

[50] Viswanathan S, Hammock BD, Newman JW, Meerarani P, Toborek M, Hennig B. Involvement of CYP 2C9 in Mediating the Proinflammatory Effects of Linoleic Acid in Vascular Endothelial Cells. Journal of the American College of Nutrition. 2003;22(6):502–10. 10.1080/07315724.2003.10719328 14684755

[51] Lynes MD, Leiria LO, Lundh M, Bartelt A, Shamsi F, Huang TL, et al The cold-induced lipokine 12,13-diHOME promotes fatty acid transport into brown adipose tissue. Nat Med. 2017;advance online publication. 10.1038/nm.4297 http://www.nature.com/nm/journal/vaop/ncurrent/abs/nm.4297.html#supplementary-information. 28346411 PMC5699924

[52] McNamara JP, Valdez F. Adipose tissue metabolism and production responses to calcium propionate and chromium propionate. J Dairy Sci. 2005;88(7):2498–507. Epub 2005/06/16. 10.3168/jds.S0022-0302(05)72927-1 . 15956312

[53] Yuan K, Farney JK, Mamedova LK, Sordillo LM, Bradford BJ. TNFα Altered Inflammatory Responses, Impaired Health and Productivity, but Did Not Affect Glucose or Lipid Metabolism in Early-Lactation Dairy Cows. PLOS ONE. 2013;8(11):e80316 10.1371/journal.pone.0080316 24260367 PMC3833956

[54] Kwon HJ, Kim SN, Kim YA, Lee YH. The contribution of arachidonate 15-lipoxygenase in tissue macrophages to adipose tissue remodeling. Cell Death Dis. 2016;7(6):e2285 Epub 2016/07/01. 10.1038/cddis.2016.190 27362803 PMC5108340

[55] Wamberg L, Christiansen T, Paulsen SK, Fisker S, Rask P, Rejnmark L, et al Expression of vitamin D-metabolizing enzymes in human adipose tissue[mdash]the effect of obesity and diet-induced weight loss. Int J Obes. 2013;37(5):651–7. 10.1038/ijo.2012.112 22828938

[56] Aitken SL, Karcher EL, Rezamand P, Gandy JC, VandeHaar MJ, Capuco AV, et al Evaluation of antioxidant and proinflammatory gene expression in bovine mammary tissue during the periparturient period. J Dairy Sci. 2009;92(2):589–98. 10.3168/jds.2008-1551 19164669

[57] Engels F, Willems H, Nijkamp FP. Cyclooxygenase-catalyzed formation of 9-hydroxylinoleic acid by guinea pig alveolar macrophages under non-stimulated conditions. FEBS Letters. 1986;209(2):249–53. 10.1016/0014-5793(86)81121-8 3098580

[58] Ramsden CE, Ringel A, Feldstein AE, Taha AY, MacIntosh BA, Hibbeln JR, et al Lowering dietary linoleic acid reduces bioactive oxidized linoleic acid metabolites in humans. Prostaglandins, Leukotrienes and Essential Fatty Acids. 2012;87(4–5):135–41. 10.1016/j.plefa.2012.08.004 PMC3467319 22959954

[59] Zha W, Edin ML, Vendrov KC, Schuck RN, Lih FB, Jat JL, et al Functional characterization of cytochrome P450-derived epoxyeicosatrienoic acids in adipogenesis and obesity. Journal of Lipid Research. 2014;55(10):2124–36. 10.1194/jlr.M053199 25114171 PMC4174005

[60] Lee JP, Yang SH, Lee H-Y, Kim B, Cho J-Y, Paik JH, et al Soluble Epoxide Hydrolase Activity Determines the Severity of Ischemia-Reperfusion Injury in Kidney. PLOS ONE. 2012;7(5):e37075 10.1371/journal.pone.0037075 22590647 PMC3349654

[61] Girousse A, Tavernier G, Valle C, Moro C, Mejhert N, Dinel AL, et al Partial inhibition of adipose tissue lipolysis improves glucose metabolism and insulin sensitivity without alteration of fat mass. PLoS biology. 2013;11(2):e1001485 Epub 2013/02/23. 10.1371/journal.pbio.1001485 23431266 PMC3576369

