# Supplementary material for: Periparturient lipolysis and oxylipid biosynthesis in bovine adipose tissues
Source: PLoS One. 2017 Dec 5;12(12):e0188621. doi: 10.1371/journal.pone.0188621 (PMC5716552; doi:10.1371/journal.pone.0188621)
Supplement: S1 File — Supplementary Tables A, B, C, and D. (DOCX) [file pone.0188621.s001.docx]

**Supplemental information**

**Table A.**

Nutrient composition of gestation and lactation diets

| **Ingredients, % DM** | G1 | G2 | PP |
| --- | --- | --- | --- |
| Grass Hay | 20.0 | 35.4 | - |
| Alfafa Hay | - | - | 11.3 |
| Corn Silage | 38.6 | 36.9 | 30.3 |
| Alfafa Silage | 19.5 | - | 15.6 |
| Wheat Straw | 11.3 | - | - |
| Ground Corn | - | 7.02 | 12.5 |
| High Moisture Corn | - | - | 6.22 |
| Soybean meal | 8.75 | 13.1 | 13.1 |
| Soyhulls | - | - | 3.11 |
| Whole Cottonseed | - | - | 3.54 |
| Mineral and Vitamin mix | 2.0 | 7.54 | 4.3 |
| **Chemical composition, % DM** |  |  |  |
| NDF | 52.1 | 44.3 | 30.5 |
| Forage NDF | 51.3 | 41.3 | 24.0 |
| CP | 11.7 | 13.8 | 17.7 |
| Starch | 16.4 | 18.5 | 24.0 |
| FA | 1.67 | 1.80 | 2.50 |
| Ca | 0.62 | 0.84 | 1.08 |
| P | 0.26 | 0.30 | 0.55 |
| FA | 1.67 | 1.80 | 2.50 |

**Table B.**

Fatty acid composition of gestation and lactation diets

|  | G1 | G2 | PP |
| --- | --- | --- | --- |
| % diet |  |  |  |
| Total FA | 1.65 | 1.82 | 3.74 |
| C16:0 | 0.16 | 0.23 | 1.10 |
| C18:0 | 0.05 | 0.06 | 0.13 |
| C18:1 | 0.25 | 0.3 | 0.61 |
| C18:2 n-6 | 0.65 | 0.76 | 1.50 |
| C18:3 n-3 | 0.05 | 0.05 | 0.18 |

**Table C.**

Taqman assays information

| Gene | Product^1^ | RefSeq |
| --- | --- | --- |
| *ARG1* | Bt03238885_m1 | NM_001046154.1 |
| *ALOX5* | Bt00386520_m1 | NM_001192792.1 |
| *ALOX15* | Bt03214775_m1 | NM_174501.2 |
| *B2M* | Bt03251628_m1 | NM_173893.3 |
| *CCL2* | Bt03212321_m1 | NM_174006.2 |
| *CCL22* | Bt 03271471_m1 | NM_001099162.2 |
| *CD44* | Bt03212355_m1 | NM_174013.3 |
| *CD68* | Bt03233854_g1 | [NM_001045902.1](http://www.ncbi.nlm.nih.gov/nuccore/NM_001045902.1) |
| *EIF3K* | Bt03226565_m1 | NM_001034489.2 |
| *EPHX2* | Bt03241449_m1 | NM_001075534.1 |
| *CYP2A1* | Bt03237496_m1 | NM_174530.2 |
| *CYP2J2* | Bt00356035_m1 | NM_001077908.1 |
| *IL10* | Bt03212727_m1 | NM_174088.1 |
| *IL6* | Bt03211905_m1 | NM_173923.2 |
| *PTGS2* | Bt03214492_m1 | NM_174445.2 |
| *RPS9* | Bt03272016_m1 | NM_001101152.2 |
| *SPP1* | Bt03213107_m1 | NM_174187.2 |
| *TNF* | Bt03259154_m1 | NM_173966.3 |

**Table D.**

Blood concentrations of albumin, fetuin A, and cholesterol in dairy cows with low (LL, FFA<1.0 mEq/L) and high (HL, FFA≥1.0 mEq/L) lipolysis rate postpartuum (PP at -27±7 (G1) and -10±5 d (G2) prepartum and at 8±3 d postpartum (PP) in cows.
